# Land cover dataset of the China Central-Asia West-Asia Economic Corridor from 1993 to 2018

**DOI:** 10.1038/s41597-023-02623-z

**Published:** 2023-10-20

**Authors:** Amin Naboureh, Ainong Li, Jinhu Bian, Guangbin Lei, Xi Nan

**Affiliations:** 1 Research Center for Digital Mountain and Remote Sensing Application, Institute of Mountain Hazards and Environment, Chinese Academy of Sciences, Chengdu, 610041 China; 2 Wanglang Mountain Remote Sensing Observation and Research Station of Sichuan Province, Mianyang, 621000 China

**Keywords:** Earth and environmental sciences, Geography

## Abstract

Land Cover (LC) maps offer vital knowledge for various studies, ranging from sustainable development to climate change. The China Central-Asia West-Asia Economic Corridor region, as a core component of the Belt and Road initiative program, has been experiencing some of the most severe LC change tragedies, such as the Aral Sea crisis and Lake Urmia shrinkage, in recent decades. Therefore, there is a high demand for producing a fine-resolution, spatially-explicit, and long-term LC dataset for this region. However, except China, such dataset for the rest of the region (Kyrgyzstan, Turkmenistan, Kazakhstan, Uzbekistan, Tajikistan, Turkey, and Iran) is currently lacking. Here, we constructed a historical set of six 30-m resolution LC maps between 1993 and 2018 at 5-year time intervals for the seven countries where nearly 200,000 Landsat scenes were classified into nine LC types within Google Earth Engine cloud computing platform. The generated LC maps displayed high accuracies. This publicly available dataset has the potential to be broadly applied in environmental policy and management.

## Background & Summary

Land Cover (LC) changes influence most natural and physical processes on Earth, such as the hydrological cycle, ecological balance, and ecosystem services^1,2^. Understanding LC types and their changes over time at different spatial scales, from regional to global, is of paramount importance for multiple disciplines, such as environmental risk assessment, global warming, and sustainability^3,4^. Remote Sensing (RS) data, with its varying spatiotemporal resolutions, has become an essential tool for monitoring LC changes^5,6^. Despite the tremendous efforts that have been conducted in the LC change mapping field, accurate knowledge on historical LC changes over large areas is still limited^7,8^.

A key part of the Belt and Road Initiative (BRI) program, the China-Central Asia-West Asia Economic Corridor (here after referred to as the Corridor) encompasses eight countries (China, Kyrgyzstan, Turkmenistan, Kazakhstan, Uzbekistan, Tajikistan, Turkey, and Iran) that mostly are situated in arid and semi-arid climates^9^. Over the last decades, the Corridor has been impacted by some of the world’s worst LC change tragedies^9^. The Aral Sea crisis and Lake Urmia shrinkage are two main examples in this regard. More specifically, the Aral Sea in Central Asia has lost over 80 percent of its surface over the past few decades^10^, and Lake Urmia in Iran has similarly shrunk in surface area by 80 percent between 2005 and 2015^11^. Given that those kinds of severe LC changes can threaten lives of millions of humans and species^12–14^ the precise long-term LC dataset along the Corridor is therefore essential for understanding the main causes of such changes.

A number of high to coarse resolutions global LC products, such as FROM-GLC^15^, GlC2000^16^, and MODIS^17^ datasets have been generated by the RS community. However, the existing LC products have limited applicability for a variety of long-term LC change monitoring applications due to their narrow temporal coverage, relatively low accuracies, and inconsistencies in the classification systems among different global LC products^7,9^. For example, although the Globland30 dataset is indeed a valuable product, theses dataset is only available at decadal intervals (2000, 2010, and 2020). It is also impractical to use this LC dataset as a benchmark for long-term LC change monitoring aim due to its unknown accuracy for 2000 and relatively low accuracy in certain regions, such as Central Asia where Sun *et al*.^18^, indicated a low Overall Accuracy (OA) of 46 percent. Similarly, Naboureh *et al*.^19^, claimed that Globland30 had a relatively low OA of 62.3 percent for Turkey. Thus, it is impractical to study historical LC changes using the available LC products.

Addressing this deficiency, the Landsat archive’s free sharing policy in 2008^2,20^ and the advent of Earth science data cloud computing and analysis tools, such as Google Earth Engine (GEE), have facilitated the production of various types of LC products^19^. As a result, several valuable LC datasets for various regions of the world have been generated. For instance, Calderón-Loor *et al*.^7^, generated a set of seven 30-m resolution LC maps covering the period of 1985–2015 at five-year intervals. In another attempt, Pekel *et al*.^21^, generated annual global surface water area dataset from 1984 to 2016. However, our team’s review^9^ revealed that a long-term LC dataset is still lacking for the majority of the Corridor, including Kyrgyzstan, Turkmenistan, Tajikistan, Uzbekistan, Iran, and Turkey. To the best of our knowledge, the only exception is China, where Yang and Huang^5^ created the first 30-m annual LC dataset spanning the period between 1990 and 2019, based on the GEE platform and Landsat data.

**Fig. 1:**
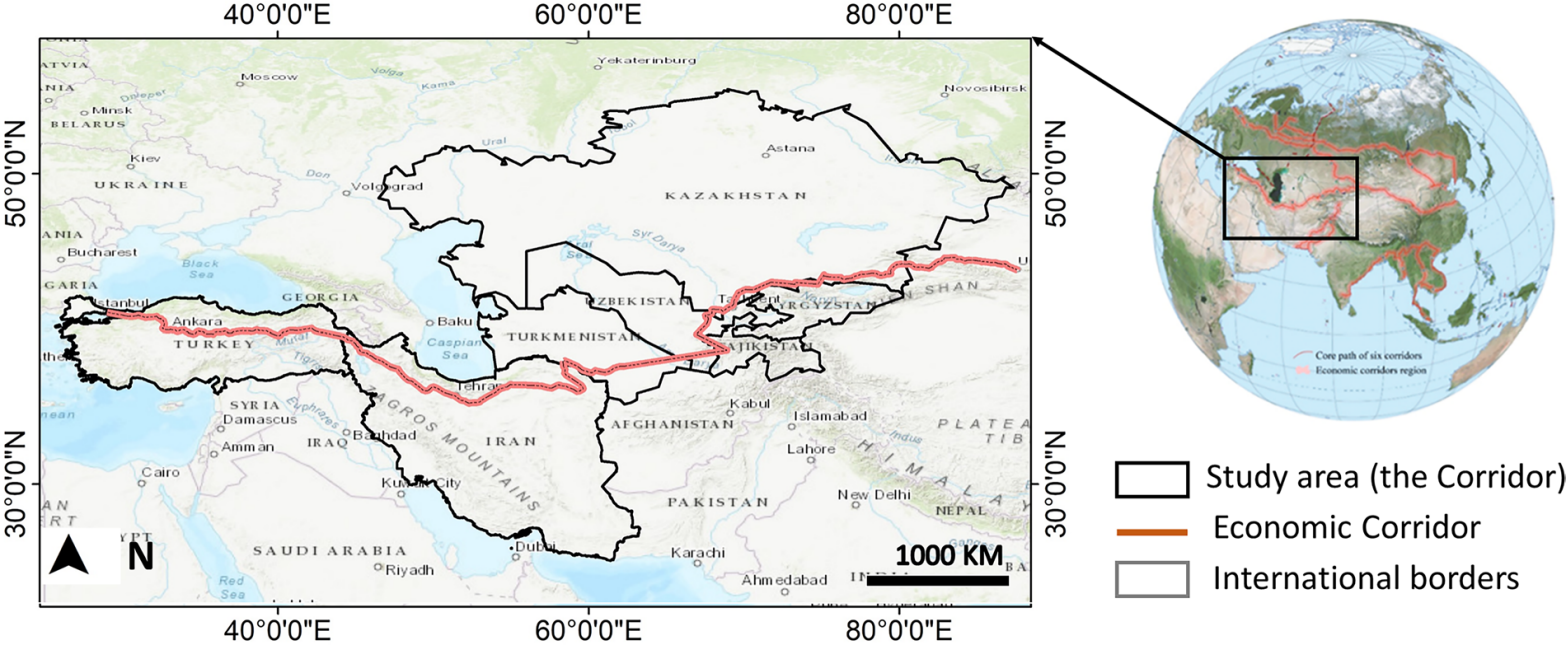
The location of the study area.

This paper presents a set of six 30-m resolution LC maps for seven countries from the Corridor, excluding China, namely Kyrgyzstan, Turkmenistan, Tajikistan, Uzbekistan, Iran, and Turkey (Fig. 1). To generate these maps, we utilized all available surface reflectance Landsat data from 1990 to 2020 through the GEE platform. Furthermore, we compared the generated LC maps against the available scientific LC products to ensure the accuracy of the results. The generated dataset is the first-of-its-kind for one of the BRI’s Corridors, filling a significant knowledge gap in the region. The dataset provides valuable information to policymakers, enabling them to make informed decisions on land use/cover management, environmental planning, and sustainable development in the region.

## Methods

**Fig. 2:**
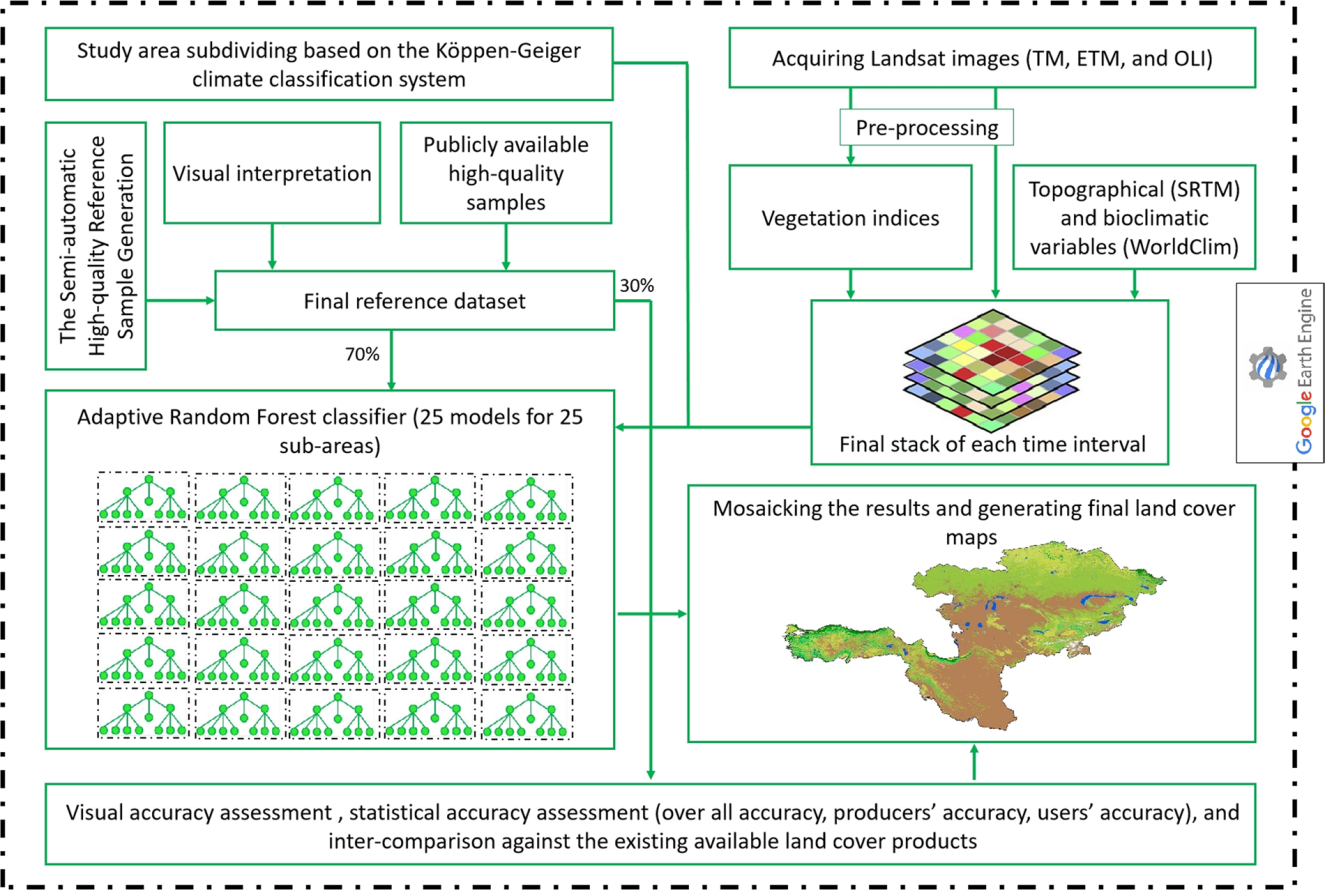
Main flowchart of this study.

This study offers a set of six 30-m LC maps for the seven countries from the Corridor between 1993 and 2018. The entire procedure was carried out on the GEE cloud processing platform (Fig. 2) that allowed us to derive pixel-wise analysis while bypassing the need for data download. The analysis was performed in 5-year epochs (1991–1995, 1996–2000, 2001–2005, 2006–2010, 2011–2015, 2016–2020). We created one LC map for per epoch (six LC maps in total); for instance, the 1993 LC map represents the 1991–1995 epoch. As shown in Table 1, the generated LC map at each time step includes nine LC classes: Bare land, Built-up, Shrub lands, Forest, Cropland, Grassland, Wetland, Snow/Ice, and Water. In general, the study consisted of six main steps (Fig. 2): 1) Study area subdivision, 2) Satellite data acquisition and pre-processing, 3) Input features generation, 4) Reference sample data collection, 5) Supervised classification, and 6) Accuracy assessment.

**Table 1:** The classification system used in this study and descriptions of each land cover type.

LC class	Definition	ID
Forest	Land covered by trees, vegetation covering more than30% of the area, such coniferous and deciduous forests, and sparse woodland.	0
Bare land	Land with less than 10% vegetation cover, such as desert, bare rocks, sandy fields, saline and alkaline land, etc.	1
Cropland	Land dedicated to farming (irrigated and dry farmland), and gardening.	2
Built-up	Land characterized by a man-made and often impervious surface of constructions and pavement, such all kinds of residence, transportation infrastructures, bridges and viaducts, solar panels, and power plants, etc.	3
Shrub lands	Land dominated (over 10%) by shrubs and low woody plants, including evergreen and deciduous shrubs, tundra, and desert steppe, etc.	4
Grassland	Land with natural grass covering more than 10% of the area, etc.	5
Snow/Ice	Lands characterized by permanent snow, ice, and glacier.	6
Wetland	Land occupied by wetland plants and water areas, such as marsh, mangrove, bogs and river floodplain wetland.	7
Water	Land covered by water bodies, such as reservoirs, lakes, streams, and rivers.	8

### Study region subdividing

**Fig. 3:**
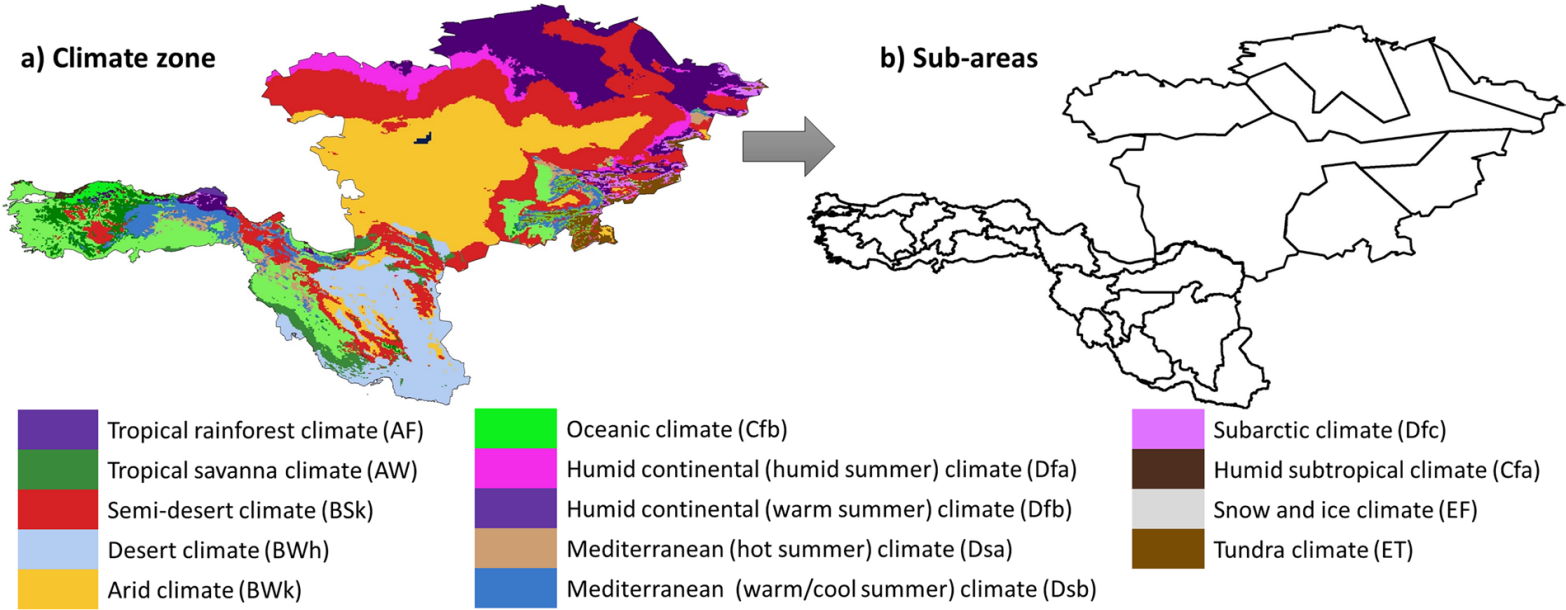
(**a**) Köppen-Geiger climate classification for the study area. (**b**) Location of introduced sub-areas.

This study focuses on five Central-Asian countries (Turkmenistan, Uzbekistan, Kyrgyzstan, Tajikistan, and Kazakhstan) and two West-Asian countries (Turkey and Iran), with a total area of about 6,435,208 km^2^. The large area covered by the study area includes a wide range of climate zones, from cold-desert climate to hot-semi-arid climate, which can lead to significant intra-class variability of spectral signatures of the same LC type and potentially reduce LC classification accuracy. On the other hand, large-scale studies require an immense size of input features, making processing of the entire region often unfeasible^22^. Our recently published research^19^ has shown that adopting an adaptive classification approach that divides the large-scale region into several sub-areas can effectively address these issues. As such, we divided the Corridor region into 25 sub-areas identified by Köppen-Geiger climate classification system^23^ (Fig. 3). It’s worth noting that we combined similar classes due to the presence of small detected zones. Furthermore, to avoid processing timeouts, certain regions were divided into two or three sub-areas. The Köppen-Geiger integrates the mean yearly and monthly temperatures and rainfall and seasonal rainfall to divide the study area into fairly homogeneous zones. The main advantages of this approach are twofold: it allowed us to bypass “user storage limited” and “computational time out” limitations of the GEE platform, while also decreasing the effect of climate variation on LC classification success.

### Satellite data acquisition and pre-processing

**Fig. 4:**
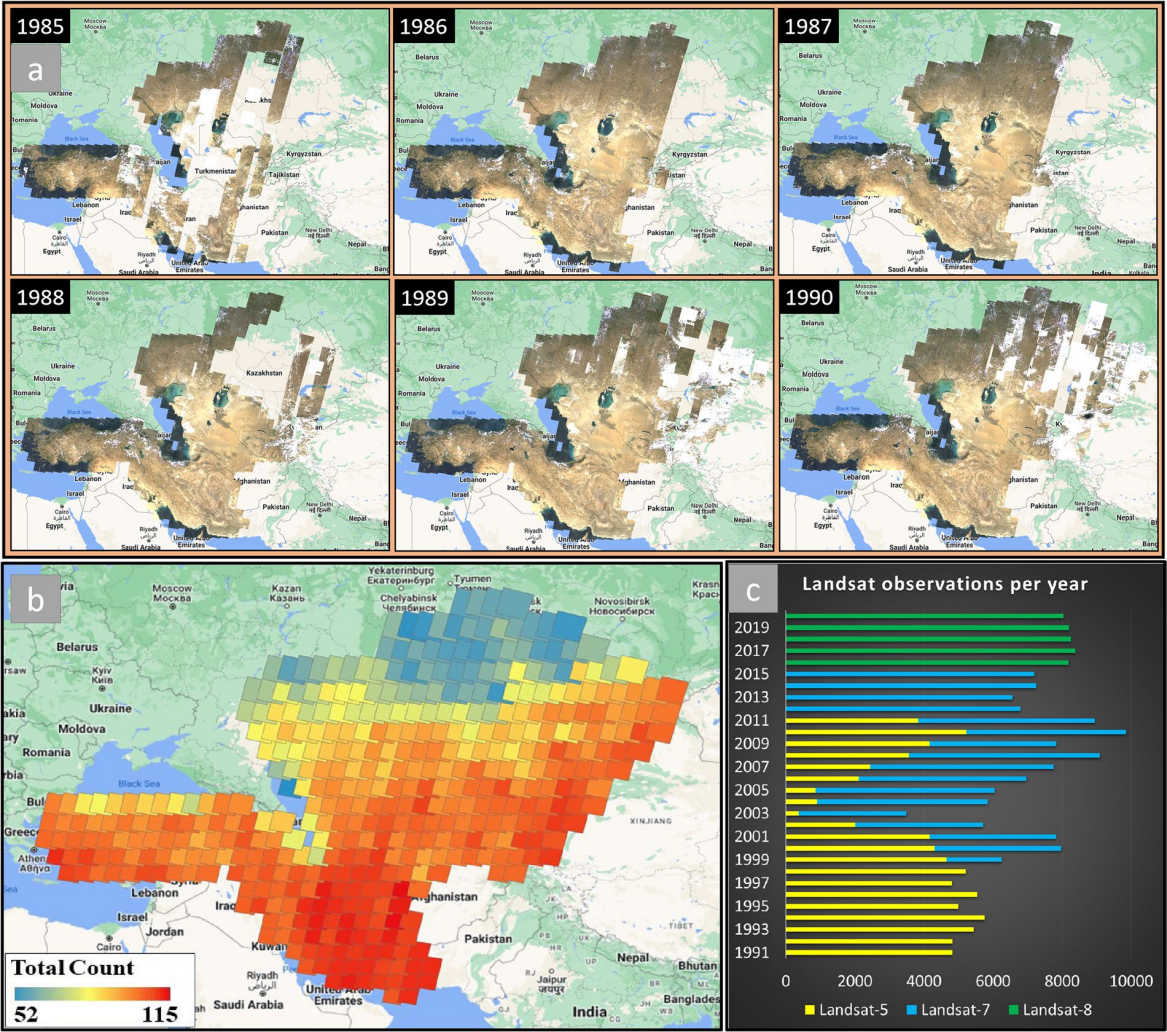
(**a**) Distribution of available surface reflectance Landsat −5 images for the years 1985, 1986, 1987, 1988, 1989, and 1990. (**b**) Observation counts from surface reflectance Landsat-8 across the study area to generate the year 2018 mosaic (1^st^ January 2016 to 31^st^ December 2020). (**c**) Number of the processed surface reflectance Landsat images in this study per year.

Since the lunch of Landsat-5 in 1984, Landsat satellites (TM, ETM+ , and OLI/TIRS) have been offering 30-m Earth observation data, which is widely known as an ideal source for LC mapping over large areas^7,9^. In this study, all the available surface reflectance Landsat data between 1^st^ January 1991 and 31^st^ December 2020, were acquired within the GEE cloud processing platform, as there were not adequate Landsat scenes covering the entire study area during 1984–1990 (Fig. 4a). A total of 194,155 Landsat scenes were obtained, including 70,339 Landsat-5, 82,770 Landsat-7, and 41,046 Landsat-8 images, to generate LC maps for the years 1993, 1998, 2003, 2008, 2013, and 2018 (Fig. 4c). To mitigate missing data and cloud interference and, a stack of Landsat scenes for the target year ± two years was created at each time-stack (e.g., times-series of Landsat-7 scenes from 1991 to 1995 were utilized to create the year 1993 mosaic), where each stack contained at least 5 valid observations at pixel-level. The FMASK algorithm^24^ was then applied to eliminate unwanted pixels, such as cloud and cloud-shadows.

### Input features generation

Six bands from Landsat data, including Short-wave infrared 1, Short-wave infrared 2, Near-infrared, Green, Blue, and Red were utilized in this work. Moreover, using the Random Forest (RF) variable selection technique, eight spectral indices were calculated among various spectral indices to contribute in LC mapping (Table 1). As shown in Table 1, along with those spectral bands and indices, bioclimatic variables (mean annual temperature and annual precipitation) and topographical features (elevation, slope, and aspect) were also created and added to the cloud-free stacks. The topographical features were taken from the Shuttle Radar Topography Mission (SRTM) data, and the bioclimatic variables were obtained from the WorldClim^25^ data. Using the nearest neighbour interpolation method, all auxiliary variables were resampled to 30-m pixels in accordance with the Landsat pixel grid. Finally, a mean function was applied to all cloud-free stacks at each time step to generate a single mosaic image for classifying each LC map.

### Reference sample data collection

We used three different techniques to collect high-quality reference samples for calibration and testing. The first method used was the Semi-automatic High-quality Reference Sample Generation method (HRSG) developed by our team^19^. This method acknowledges that no scientific LC product/map can be entirely free of errors, and a limited number of training samples are capable of representing intra-class diversity. Consequently, the HRSG method acquires the identified pixels of the target LC type from the existing LC products, then implement the linear spectral unmixing analysis to obtain samples for the target class. In this study, a wide range of existing LC products, such as GlobLand30^26^, GLC-FCS30^27^, MCD12Q1^17^,Global Artificial Impervious Area (GAIA)^28^, and JRC global surface water^21^, were used to obtain reference sample datasets for nine LC classes at five different time-steps (Table 3).

**Table 2:** Ancillary variables used in the classification model.

	Abbrev.	Description	Formula
Spectral values	SWIR2	Short-wave infrared 1	
	SWIR1	Short-wave infrared 2	
	NIR	Near infrared	
	R	Red	
	G	Green	
	B	Blue	
Vegetation indices	NDVI^36^	Normalized difference vegetation index	$\frac{{\rm{NIR}}-{\rm{R}}}{{\rm{NIR}}+{\rm{R}}}$
	BSI^37^	Bare soil index	$\frac{\left({\rm{SWIR}}2+{\rm{R}}\right)-\left({\rm{NIR}}+{\rm{B}}\right)}{\left({\rm{SWIR}}2+{\rm{R}}\right)+\left({\rm{NIR}}+{\rm{B}}\right)}$
	SATVI^38^	Soil-adjusted total vegetation index	$1.5\times \frac{{\rm{SWIR}}1-{\rm{R}}}{{\rm{SWIR}}+{\rm{R}}+0.5}+\frac{{\rm{SWIR}}2}{2}$
	NDBI^39^	Normalized difference built-up index	$\frac{{\rm{SWIR}}-{\rm{NIR}}}{{\rm{SWIR}}+{\rm{NIR}}}$
	MSAVI^40^	Modified Soil-adjusted vegetation index	$\frac{2\times \mathrm{NIR}+1-\sqrt{{\rm{B}}(2\times \mathrm{NIR}+1){}^{2}\,-\,8\times (\mathrm{NIR}-{\rm{R}})}}{2}$
	NBR^41^	Normalized burn ratio	$\frac{{\rm{NIR}}-{\rm{SWIR}}2}{{\rm{NIR}}+{\rm{SWIR}}2}$
	EVI^42^	Enhanced vegetation index	$2.5\times \frac{\mathrm{NIR}-{\rm{R}}}{\mathrm{NIR}+6\times \mathrm{Red}-7.5\times {\rm{B}}+1}$
	DMSP	Night-time stable lights	
Other		Mean yearly precipitation	
		Mean yearly temperature	
		Aspect	
		Slope	
		Elevation

**Table 3:** Land cover products used to generate high-quality reference sample dataset in this study.

Land cover product	Time	Resolution	2018	2013	2008	2003	1998	1993
Globland-30^26^	2000, 2010, 2020	30 m	√	√	√	√	√	√
MCD12Q1^17^	2001–2020	500 m	√	√	√	√	√	√
JRC global surface water^21^	1985–2020	30 m	√	√	√	√	√	√
GAIA maps^28^	1985–2018	30 m	√	√	√	√	√	√
GLC-FCS30^27^	2015	30 m	√	√	×	×	×	×
FROM-GLC30^15^	2017	30 m	√	√	×	×	×	×

In order to ensure an adequate number of samples and a diverse representation of each LC class (random sampling technique), the visual interpretation technique was used when the collected samples by HRSG method was insufficient. The study aimed to have at least 30,000 training samples at each time step to achieve acceptable accuracy according to the conducted initial experiments. Therefore, samples were obtained using Landsat images, annual NDVI time series, and the Google Earth images. More specifically, following Naboureh *et al*.^19^, the samples were first collected using Google Earth (publicly available fine spatial resolution images) and Landsat images. Then, their annual NDVI time series were extracted from Landsat images. Finally, the true label was determined for an LC class if the NDVI values of the given sample was stable over each time step.

Additionally, the available high-quality samples generated by RS community was used in this study. One such example is the cropland data in Central Asia, which has been provided by Remelgado *et al*.^29^, Their dataset includes a total of 8,196 samples collected between 2015 and 2018, as well as 213 samples from 2011 and 26 samples from 2008. We double checked these samples, and stable samples were added to the generated dataset for 2013 and 2018. Overall, the final dataset for each time interval includes over 50,000 pixel points (Table 4) that is split into a training (70%) and validation (30%). Table 4 presents the proportion of samples for each LC type in 2018.

**Table 4:** Number of samples and area for each land cover type for the year 2018.

Type	Number of Samples	(%)	Type	Number of Samples	(%)
Forest	5177	9.1	Grass	9515	17
Bare	11887	21	Snow/Ice	874	1
Cropland	9794	17.3	Wetland	1926	3
Built-up	6858	12.1	Water	5374	9
Shrub	5060	9	Total = 56465		

We conducted initial experiments to eliminate the possibility of discontinuous classification results near climatic zone boundaries, as reported by Shafizadeh-Moghadam *et al*.^22^, We observed instances of sudden fluctuations occurred near climate zone boundaries when the distribution of training samples was not well-balanced. To address this challenge, we adopted a rigorous technique to ensure that training samples are evenly (to the greatest extent possible) distributed across all sub-areas. This not only improved the model’s ability to generalize in diverse regions but also lowered the risk of discontinuous classification results across climatic zone boundaries.

### Supervised classification

There is a broad selection of supervised LC classification algorithms available, such as neural network, maximum likelihood classification, support vector machine, extreme learning machines, and RF. However, there is no final recognition on introducing a single classifier that offers the most precise outputs in all landscapes and circumstances yet because the given model setting and environmental conditions vary among different study areas^9,30^. RF has become a popular choice for large-scale LC classification due its ability to handle complex and high-dimensional data^31^. An investigation, which focused on six different landscapes in the Corridor, found that RF had the best performance among several well-known supervised classification algorithms^32^. In addition, our research^19^ revealed that using an adaptive RF-based classification method can effectively address the impact of climate variations on LC classification accuracy. Consequently, to create LC maps for the years 1993, 2003, 2008, 2013, and 2018, this study utilized an adaptive RF classification scheme. The study region was separated into 25 sub-areas (as shown in Fig. 3), and a separate RF classifier was developed for each sub-area. The results of each sub-area were then combined to create the ultimate LC map for each time interval. It is worth mentioning that after testing various tuning settings, we found that adjusting the number of variables and decision trees to the square root of the number of variables and 250, respectively, yielded the best results.

### Accuracy assessment and comparison against existing LC products

We evaluated the produced LC maps using the confusion matrix to calculate three commonly used accuracy metrics: OA, F1-score, Producer Accuracy (PA), and User Accuracy (UA). In general, we used minimum 15,000 validation samples at each time interval for assessing the produced LC maps. We also used a visual interpretation technique to ensure the generated LC maps are noise free, particularly across climate zone boundaries, where sudden fluctuations can be noticed if the distribution of training reference samples is poor. Additionally, taking the year 2018 as an example, we compared the generated LC map in this study against GlobLand30 (30-m resolution) and Dynamic World^33^ (10-m resolution) products for a more comprehensive performance evaluation. To this end, the GlobLand30 version 2020 and Dynamic World version 2018 (time filter = between 1^st^ January 2018 and 31^st^ December 2018) products have been used in the comparison task. It should be noted that we focused on Turkey as testbed in this section. The reason for the selection of Turkey was twofold: Turkey encompasses different climate zones and elevation ranges, and, most importantly, there were available independent validation samples with a total of almost 6000 data points. It is worth mentioning that although the selected LC products share almost the same classification system, based on the classification system of this research (Table 5), we reclassified GlobLand30 and Dynamic World products.

**Table 5:** Reformatting the classification system of Dynamic World and GlobLand30 to correspond to the classification system of the current study.

Present study	Dynamic World	GlobLand30
Forest	Trees	Forest
Bare land	Bare	Tundra & Bare land
Cropland	Crops	Cultivated Land
Built-up	Built	Artificial Surfaces
Shrub land	Shrub and scrub	Shrub land
Grasslands	Grass	Grasslands
Wetlands	Flooded vegetation	Wetlands
Snow and ice	Snow and ice	Snow and ice
Water bodies	Water	Water bodies

## Data Records

The generated data are accessible through National Tibetan Plateau Data center (10.11888/Terre.tpdc.300482)^34^. This dataset consists of six land cover maps (1993, 1998, 2003, 2008, 2013,and 2018) for the Corridor region, which spans seven countries including Turkey, Iran, Kyrgyzstan, Kazakhstan, Tajikistan, Turkmenistan, and Uzbekistan. These LC maps include nine LC classes (see Table 1), and cover the time period from 1993 to 2018. This dataset^34^ is provided at a 30 m spatial resolution in TIFF format.

## Technical Validation

To evaluate the generated map, we used visual interpretation, statistical accuracy assessment, and inter-comparison with existing LC products for a fair and comprehensive evaluation of the produced LC maps.

### Visual interpretation and statistical accuracy assessment

**Fig. 5:**
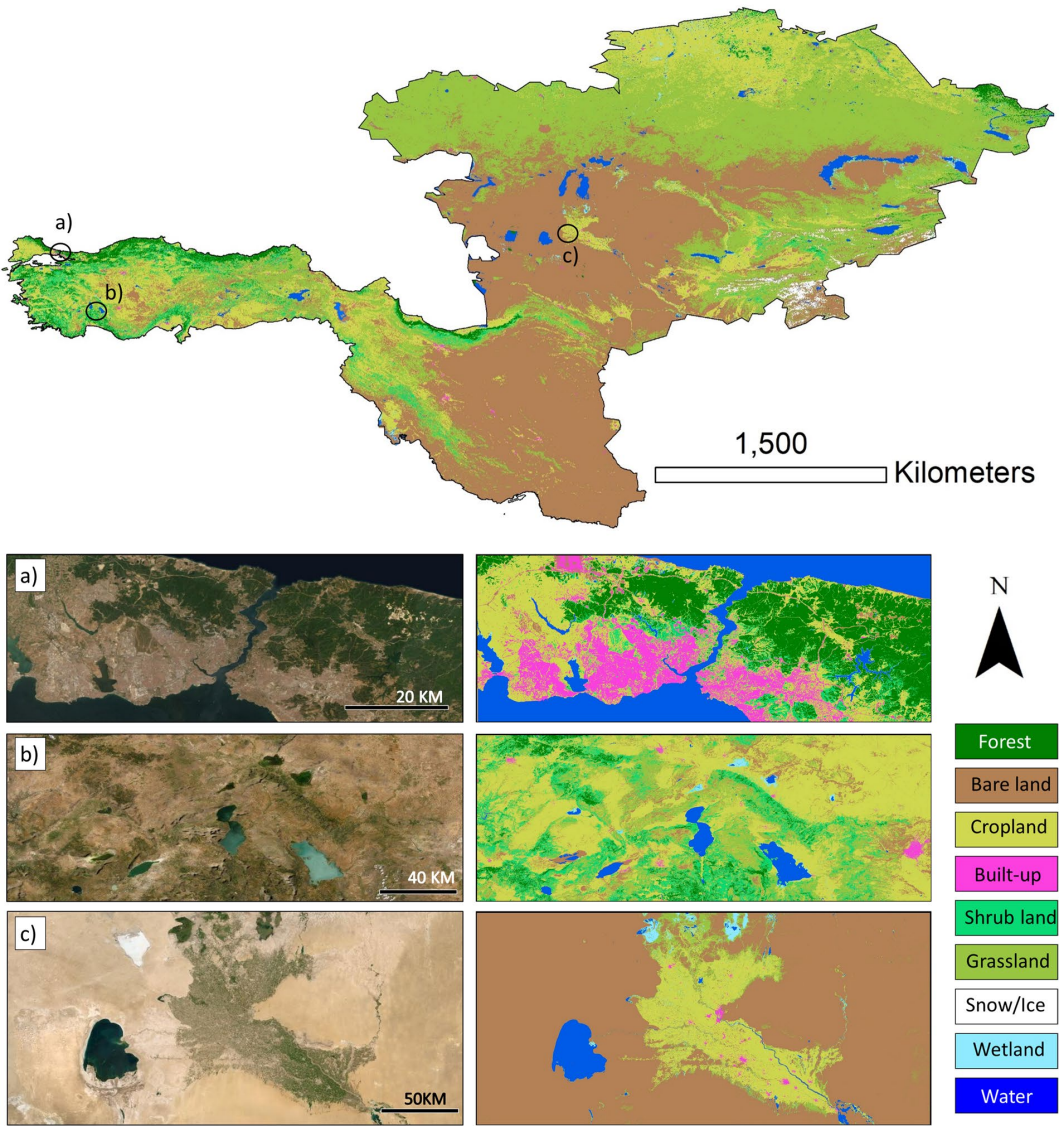
An example of the generated land cover maps for the Corridor region (year 2018), and three zoomed areas (with different climate conditions and landscapes) of the LC map along with matching Landsat images (annual median compositions).

**Fig. 6:**
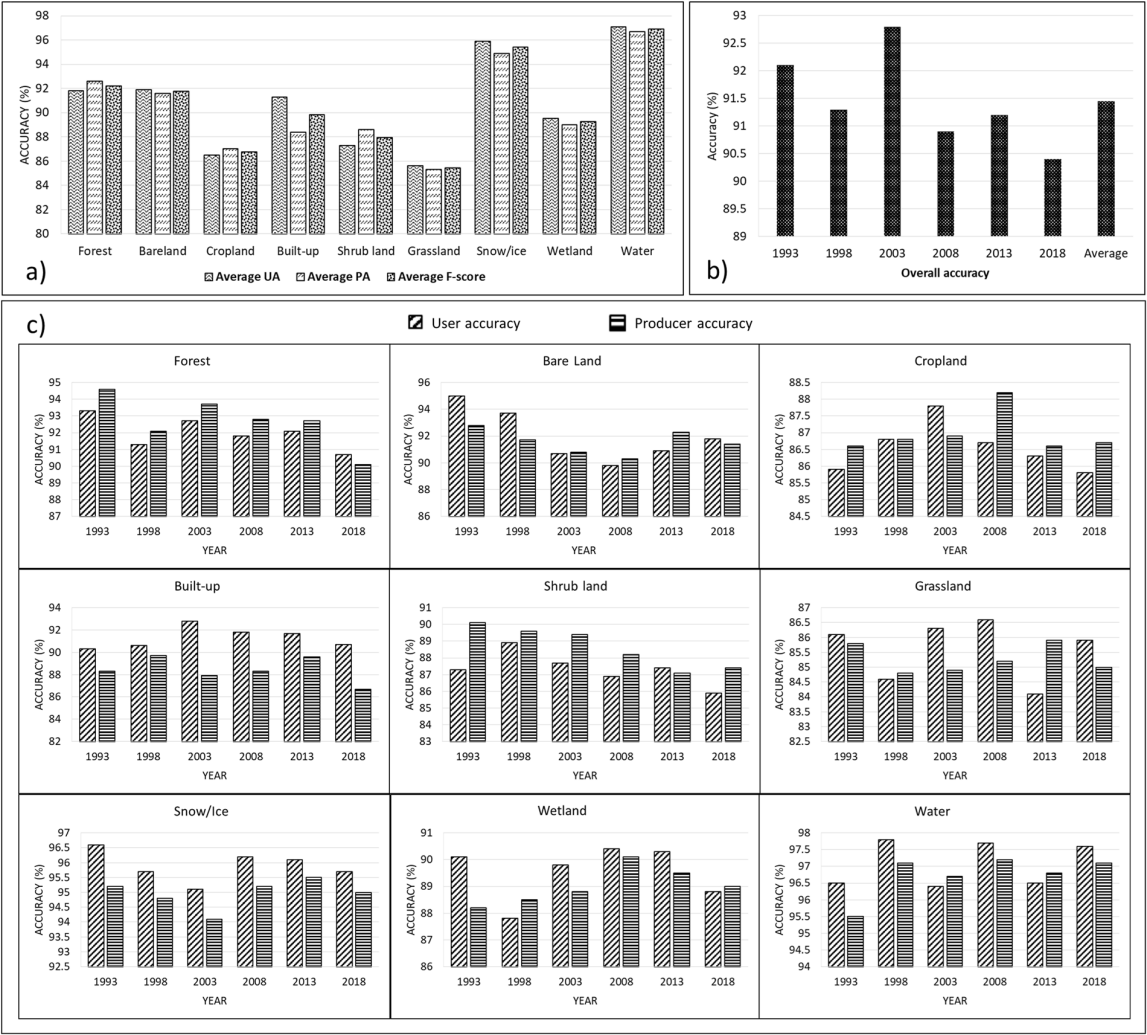
Accuracy assessment results for the generated land cover dataset. (**a**) Average user accuracy (UA), producer accuracy (PA), and F1-score accuracy per class; (**b**) Overall accuracy (OA) results at each time-step and average OA; (**c**) The PA and UA results for each land cover type at each time-step.

The visual interpretation assessment method illustrated that the generated LC dataset^34^ for the years 1993, 1998, 2003, 2008, 2013, and 2018 are noise-free and offer reasonable depictions of all nine LC types (Fig. 5). Additionally, we used minimum 15,000 (randomly selected samples) ground trust samples to implement pixel-wise validation at each time step. To this end, we calculated OA, UA, PA, and F-1 score metrics, as indicator of the performance the generated LC maps. The average OA of the generated LC maps was 91.4%, with the highest OA of 92.7% observed in 2003 and the lowest OA of 90.3% in 2018 (Fig. 6b). The accuracy of the LC classes varied considerably (Fig. 6c). As shown in Fig. 6b, Water and Snow classes displayed the highest mean PA, UA, and F1-score values (>95%) for the generated maps, while the Grassland and Cropland had lowest mean UA, PA, and F1-score values (>85%).

### Inter-comparison with existing LC products

**Fig. 7:**
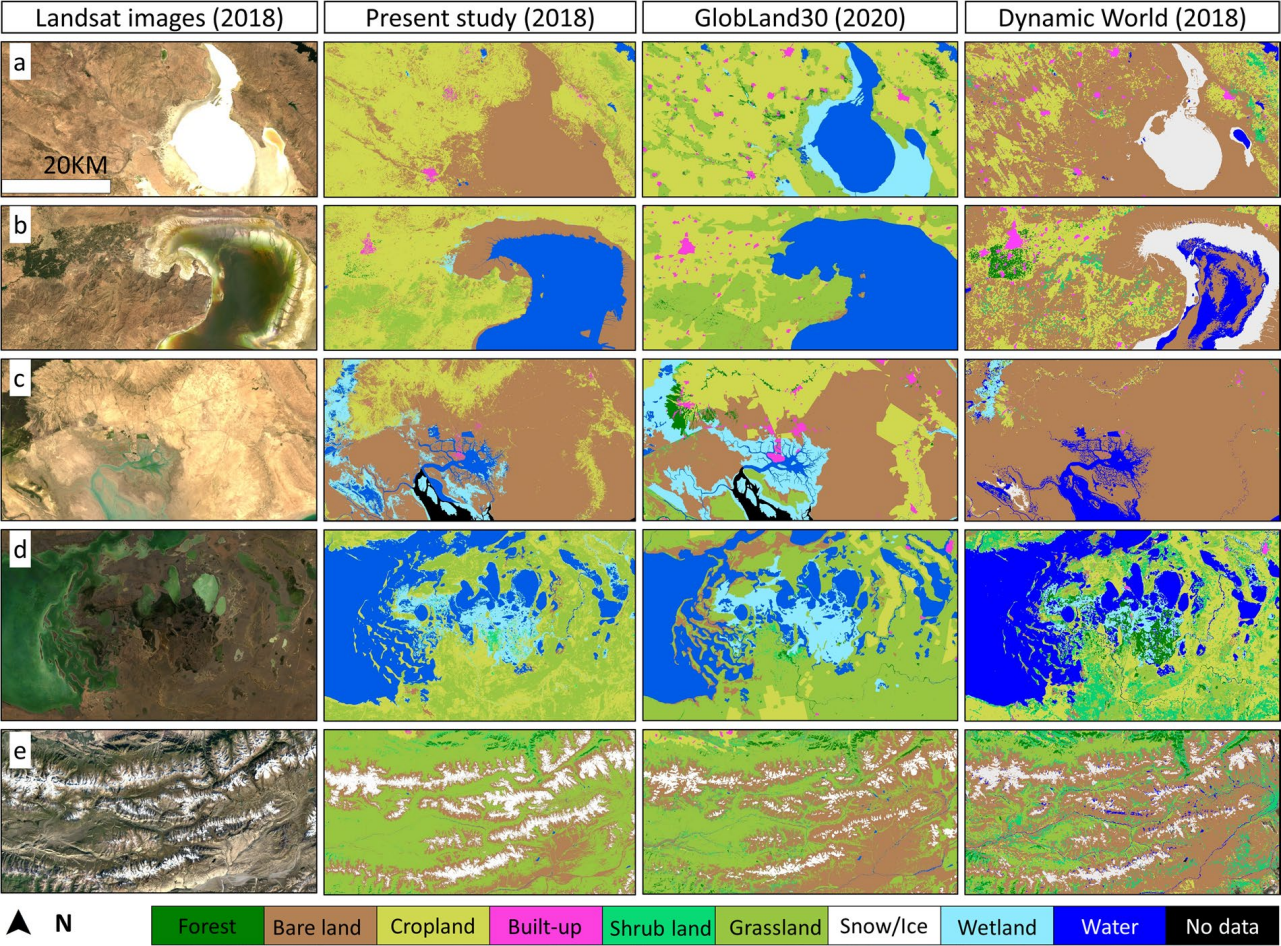
Comparison of the generated LC map of the year 2018 against GlobLand30 and Dynamic World products. The corresponding Landsat imagery are the annual median compositions of Landsat images in 2018 (1st January 2018 to 31st December 2018). The experiment sites are located (center location) at: (**a**) 38° 45′ 47″ N – 33° 21′ 03″ E; (**b**) 38° 05′ 48″ N – 45° 15′ 38″ E; (**c**) 30° 31′ 07″ N – 49° 07′ 33″ E; (**d**) 50° 23′ 42″ N – 69° 14′ 16″ E; (**e**) 41° 50′ 32″ N – 77° 22′ 21″ E.

We compared the generated LC map in this study (the year 2018) against GlobLand30 and Dynamic World Products. To this end, because GlobLand30 is not accessible for 2018, the 2020 version of GlobLand30 product was used in this study. Moreover, the mean image (1st January 2018 to 31st December 2018) of Dynamic World in 2018 were obtained from GEE platform. As can be seen in Fig. 7, our map closely reflects the exact LC type in satellite scenes. Our visual assessment showed that the generated LC map also has good consistency with the GlobalLand30 product although the generated LC map in this study could distinguish Grassland, Water, Snow, and Wetland classes more precisely than the GlobLand30 product. For instance, Fig. 7a–c shows that GlobLand30 overestimates Grassland classes, and Fig. 7d illustrates an overestimation of the wetland class. The overall layouts of all nine LC classes were almost the same for the generated LC map in this study and Dynamic World. However, the produced LC map in this work outperformed this product in distinguishing Water and Snow classes. Our visual assessment showed that the Dynamic World product misclassified the dried lake’s bed (Fig. 7a,b) as snow class. Additionally, Dynamic World mislabelled clouds and cloud shadows as Water class (Fig. 7c). In contrast, Dynamic World could distinguish built-up class more precisely than generated LC map in this study that could be related to the higher spatial resolution (10-m) of input data for this product.

**Fig. 8:**
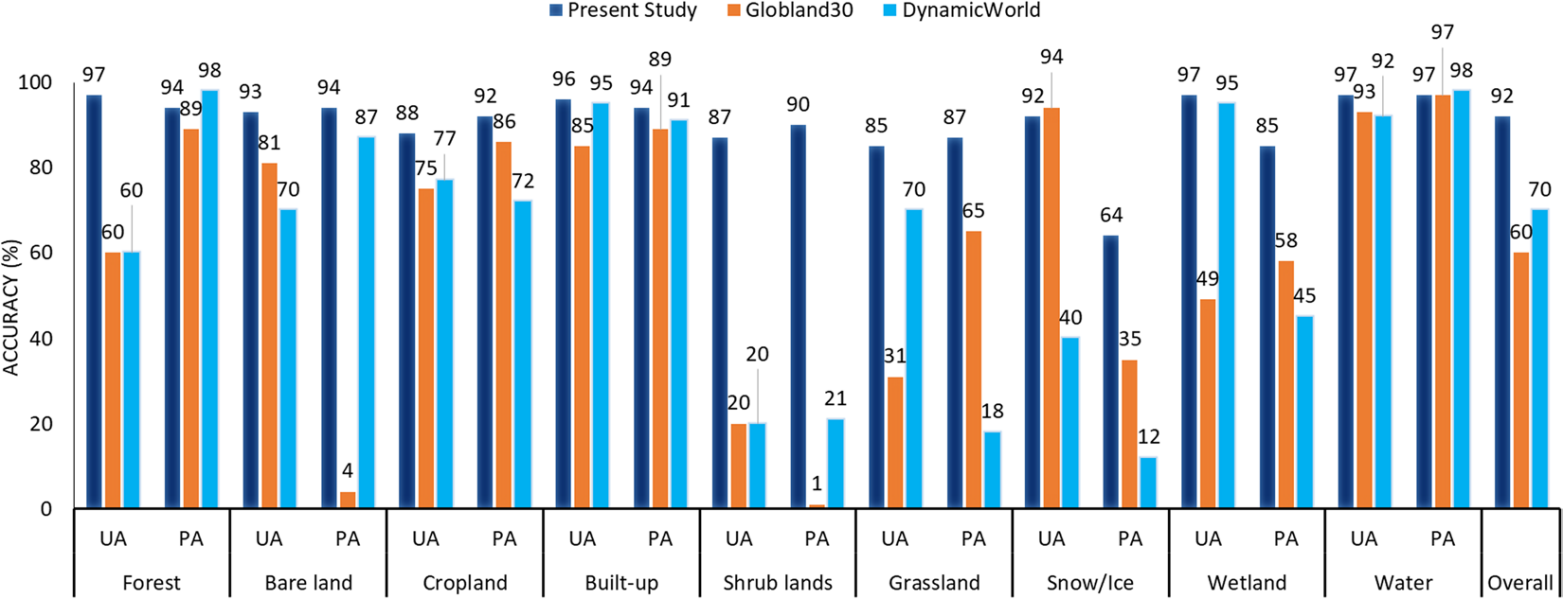
Comparison in three accuracy assessment metrics among the generated LC map in this study for the year 2018 and Globland30 (2020 version) and DynamicWorld products.

We also applied a quantitative comparison between the results of the present work with the two LC products selecting Turkey as the experiment site. It was observed that the generated LC map in this study in 2018 achieving OA = 92% outperformed Dynamic World (OA = 70%) and Globland30 (OA = 60%) products. Except for water class (PA value) in Dynamic World, snow (UA value) and cropland (UA values) classes in Globland30, the obtained PA and UA values for all nine LC classes of the generated LC map in this study were higher than the other two LC products (Fig. 8). Overall, the generated LC map in this study showed high statistical and visual accuracies that confirms the necessary confidence in the reliability of generated LC dataset. It is crucial to highlight that even though both Globland30 and DynamicWorld products showed relatively low accuracies, the enduring value and knowledge offered by these two well-known LC products remain undeniable, delivering remarkable insights to the RS community.

### Uncertainty analysis

We implemented an adaptive supervised classification scheme to accurately discriminate nine LC classes for a large-scale study. As shown in Fig. 7, our classification performed better for Snow, and Water classes whereas Grassland and Cropland classes presented a lower accuracies. We have found that the confusion between Cropland areas and Grassland/Shrub lands were common based on the conducted visual assessment. The higher accuracy for Snow and Water classes was reasonable due to their distinctive spectral response and the NDVI index’s high ability in extracting these classes. The main sources of lower accuracies for Grassland and Cropland classes could be related to: 1) spectral similarity among vegetation classes; 2) selection of ancillary variables; and, 3) Sensor limitation. Using different phonologically relevant periods and seasons (e.g., growing season) could improve the discrimination among spectrally similar classes (e.g., Cropland, Shrub land, and Grassland). However, we could not generate seasonal cloud-free Landsat data due to the lack of surface reflectance Landsat-5 data before 2000 and the issue of Landsat-7 before 2012. Different input features can greatly impact the classification results, and this study used the same nineteen variables (Table 2) as input features for the classification of 25 sub-areas at six different time steps based on the RF feature selection method. Utilizing more variables and adopting specific variables for each sub-area may lead to higher classification accuracy, but we have used a similar input feature in the classification tasks due to the input-feature limitation of GEE and the work’s time-consuming nature. The 30-m spatial resolution of Landsat data restricted the ability to distinguish LC types smaller than this scale, in particular small Cropland and Built-up areas. In general, the dataset^34^ is not error-free and the users should consider possible error/noise when interpreting the LC maps, but the generated LC dataset is reasonable.

## Usage Notes

**Fig. 9:**
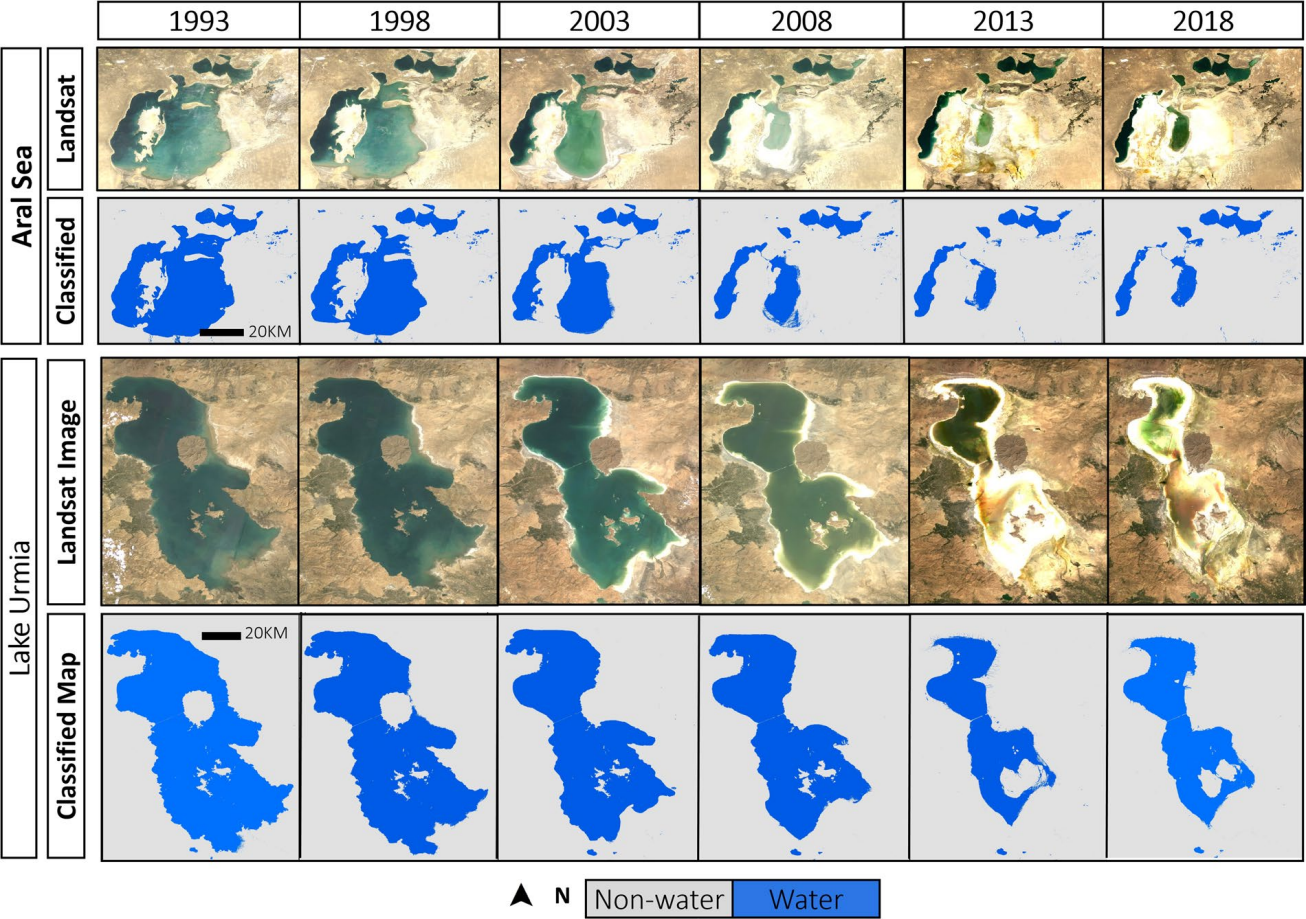
Application of the generated dataset for studying surface water changes in the region. Changes in surface water areas in two dying lakes during the 1993–2018 period: Aral Sea, Central Asia (center location = 45° 05′ 30″ N – 59° 28′ 23″ E) and Lake Urmia, Iran (center location = 37° 43′ 57″ N – 45° 21′ 40″ E).

Accurate knowledge on LC types and their changes over time is critical for different research fields, such as ecological protection, environmental management, and sustainable development. LC datasets with high spatial and temporal resolution are critical for monitoring changes in land use, identifying patterns of deforestation and urbanization, and evaluating the impact of human activities on natural ecosystems. The generated LC dataset in this study with a 30-m resolution is the first of its kind for one of the BRI’s Corridor. This dataset provides a comprehensive picture of LC dynamic for seven countries in the region since 1993. The dataset has been meticulously validated to ensure its accuracy and reliability. This dataset can be included as part of the digital BRI program, which seeks to promote the use of science and technology for sustainable development of the BRI region. This dataset has multiple applications; for example, given the remarkable shrinkages in surface water area in the Corridor region^14,21,35^ over the last few decades, affecting the lives of nearly two billion people, this dataset can serve as a useful tool for analysing the dynamic of surface water changes in the study area (Fig. 9) and identifying the main driving factors. Additionally, the dataset can be used as a baseline for predicting future status of LC in the region, enabling us to make informed and strategic choices to mitigate the adverse environmental consequences of LC alterations in the region. By sharing this dataset, we can facilitate data-driven decision-making, promote transparency and accountability, and encourage collaboration among the BRI countries.

## Data Availability

The used scripts to implement our adaptive classification scheme is available in the following link: https://github.com/AminNaboureh/Adaptive_LC_classification.git.
